# Tracking Health, Performance and Recovery in Athletes Using Machine Learning

**DOI:** 10.3390/sports10100160

**Published:** 2022-10-19

**Authors:** Denis V. Petrovsky, Vasiliy I. Pustovoyt, Kirill S. Nikolsky, Kristina A. Malsagova, Arthur T. Kopylov, Alexander A. Stepanov, Vladimir. R. Rudnev, Evgenii I. Balakin, Anna L. Kaysheva

**Affiliations:** 1 Biobanking Group, Branch of Institute of Biomedical Chemistry “Scientific and Education Center”, 109028 Moscow, Russia; 2 State Research Center–Burnasyan Federal Medical Biophysical Center of Federal Medical Biological Agency, 119435 Moscow, Russia

**Keywords:** personalized sports medicine, recovery, random forest, catabolism, anabolism

## Abstract

Training and competitive periods can temporarily impair the performance of an athlete. This disruption can be short- or long-term, lasting up to several days. We analyzed the health indicators of 3661 athletes during an in-depth medical examination. At the time of inclusion in the study, the athletes were healthy. Instrumental examinations (fluorography, ultrasound examination of the abdominal cavity and pelvic organs, echocardiography, electrocardiography, and stress testing “to failure”), laboratory examinations (general urinalysis and biochemical and general clinical blood analysis), and examinations by specialists (ophthalmologist, otolaryngologist, surgeon, cardiologist, neurologist, dentist, gynecologist (women), endocrinologist, and therapist) were performed. This study analyzed the significance of determining the indicators involved in the implementation of the “catabolism” and “anabolism” phenotypes using the random forest and multinomial logistic regression machine learning methods. The use of decision forest and multinomial regression models made it possible to identify the most significant indicators of blood and urine biochemistry for the analysis of phenotypes as a characterization of the effectiveness of recovery processes in the post-competitive period in athletes. We found that the parameters of muscle metabolism, such as aspartate aminotransferase, creatine kinase, lactate dehydrogenase, and alanine aminotransferase levels, and the parameters of the ornithine cycle, such as creatinine, urea acid, and urea levels, made the most significant contribution to the classification of two types of metabolism: catabolism and anabolism.

## 1. Introduction

Balancing between training and competitive loads and recovery is important for athletes to achieve maximum performance [1]. The endurance of athletes largely depends on the effective recovery of the body after the competition. Active endurance sports have physiological, immunological, and metabolic effects on the bodies of athletes [2]. Restoration refers to the process in which an altered biological system, including the metabolome, returns to its original state [3]. Endurance athletes are characterized by higher intensities of exercise and training due to modified muscle metabolism. As training levels are different in endurance and non-endurance athletes, exercise performance is also different, because endurance sports are characterized by repeated contractions of skeletal muscle. Typical endurance sports include cross-country, long-distance swimming, cycling, skiing, and long-distance track events, whereas non-endurance events include baseball, tennis, volleyball, softball, and short-distance/sprint events for track/field and swimming [4]. In athletes, metabolic recovery occurs in two stages: a fast stage, in which oxygen, ATP, and phosphocreatine are replenished, and a subsequent slow stage, in which the adaptations of innate metabolism are restored [1,3]. 

Training and competitive periods can temporarily impair athlete performance. This disturbance can be short-term (several minutes or hours after exercise) or long-term, lasting up to several days [1]. Recovery depends on glycogen replenishment, usually within 24 h of strenuous exercise [5], and rehydration [6]. Long-term impairment may be due to muscle damage or delayed muscle soreness (DOMS) [7]. The mechanisms mediating athlete recovery are not entirely clear; however, an imbalance between exercise stress and recovery over extended periods of time can affect the performance of an athlete. This imbalance can have long-term debilitating consequences in the form of overtraining [1].

Increased fatigue also increases the risk of injury in athletes. To accelerate the recovery process, athletes often perform structured post-competition recovery sessions. These sessions are designed to maintain the balance between exercise-induced stress and recovery. Therefore, monitoring the effectiveness of the recovery period is crucial for each athlete to take additional and timely measures to correct this period, if necessary.

Under prolonged loads, catabolic processes are mainly activated, allowing the quick and effective mobilization of the body’s energy resources to achieve a sports-related result. In contrast, anabolic processes predominate during the recovery period, allowing compensation for the loss of the body [8].

Increasingly, biomedical research is using machine learning techniques to analyze large sets of clinical data to predict the health of the human body [9]. Many studies demonstrate the promise of using machine learning methods as an auxiliary tool to help in making a clinical decision. The traditional analysis and interpretation of the results of clinical observations is a laborious process, often depending on the practical experience of the doctor. Today, the literature is actively discussing approaches to the application of machine learning methods for the classification and diagnosis of multifactorial diseases, such as coronary heart disease, cancer, diabetes mellitus and many other pathologies [9,10,11,12,13]. It is important to use the intellectual analysis of large clinical data on the health status of athletes, for which there is a significant shift in the generally accepted reference ranges of clinical indicators compared to those obtained for a sedentary population, generally reflecting the body’s adaptations to regular and prolonged physical activity.

Despite the actively developing field of data mining, there is no “gold” standard in methodological approaches yet. Depending on the task and the dimension of the dataset under study, the most popular machine learning approaches are logistic regression, support vector classifier, decision tree, multinomial naive Bayes, random forest, and multinomial regression [10,11,14,15].

The goal of this study is to examine the effectiveness of the recovery period of elite athletes in the post-competitive period. This study involved 3661 athletes who completed the competitive period and underwent an in-depth medical examination (IME). The IME of an athlete was performed to obtain complete and comprehensive information about the physical state of the body, assessing the state of health, the functional state of the body, and indicators of its physical performance. Health monitoring was performed according to the anthropometric and biochemical data by classifying the phenotype according to the type of “catabolism” and “anabolism”.

The design of a study in the absence of a generally accepted approach for analyzing the results of a clinical trial traditionally includes procedures for substantiating the choice of a machine learning method, a comparative analysis of the use of two or more methods to solve a biomedical classification problem, and assessing the reliability of the results obtained. 

Our study is built according to the traditional scheme and is aimed primarily at identifying the most important “predictors”, or clinical indicators, for assessing the health status of a professional athlete. The training process and diet are especially important for the possible adjustment of the recovery process. Random forest and multinomial logistic regression machine learning algorithms were used to classify catabolic or anabolic phenotypes.

## 2. Materials and Methods

### 2.1. Ethics Statement

All participants were informed of the risks and discomfort associated with the investigation and signed a written consent form to participate. The study was approved by the Board for Ethical Questions in A. I. Burnazyan State Research Center of the FMBA of Russia (Protocol No. 40 from 18 November 2020) according to the principles expressed in the Declaration of Helsinki.

### 2.2. Subjects

Healthy and trained athletes (*n* = 3661) participated in this study. Participants were excluded if they had a history of muscle disorder, cardiac or kidney disease, or those taking medicine (including anti-inflammatory drugs, antibiotics, and supplements) or nicotine. Each participant completed a questionnaire on their medical history and previous training. The baseline characteristics selected for this investigation are shown in Table 1.

At the time of inclusion in the study, the athletes were considered healthy based on the results of a previously completed in-depth medical examination, which included instrumental (fluorography, ultrasound examination of the abdominal cavity and pelvic organs, echocardiography, electrocardiography, and stress testing “to failure”) and laboratory examinations (general analysis of urine and biochemical and general clinical analysis of blood), as well as examinations by specialists (ophthalmologists, otolaryngologists, surgeons, cardiologists, neurologists, dentists, gynecologists (women), endocrinologists, and therapists). During the recovery period, athletes did not take pharmacological drugs or biologically active additives that could affect the biochemical analyses of the body. The physical activity took place once a day for two hours, six days a week.

The identification of the predominance of catabolic or anabolic processes (classification of four comparison groups in Table 1, column “Phenotype”) in the metabolic mechanisms of the body during the annual macrocycle was based on generally accepted (in elite sports) biomarkers of blood serum, clinical picture, and concentration of hormones of the hypothalamic–pituitary–adrenal axis (Supplementary materials Table S1). 

The severity of catabolic or anabolic regulatory mechanisms is determined by the intensity and duration of physical activity. These changes in the body of athletes are combined with high activity of the hypothalamic–pituitary–adrenal axis and are confirmed by an increase in the blood concentrations of catabolic products of protein and amino acid compounds, lipid peroxidation, and nucleic acids. Intense and prolonged physical activity is accompanied by changes in the rate of regulation of the body’s metabolism and biochemical changes in the blood plasma, confirming the violation of metabolic processes in muscle tissues and internal organs.

**Figure 1:**
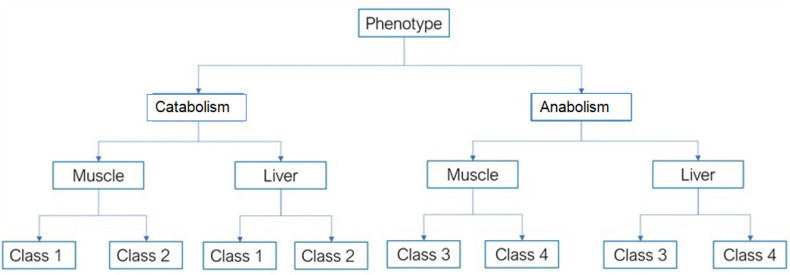
Structure of comparison groups. To classify study participants into anabolism and catabolism phenotypes, the metadata were ranked into four classes that characterize catabolism (classes 1 and 2) in the muscle and liver and anabolism (classes 3 and 4) also in the muscle and liver.

The first class was characterized by an absolute predominance of catabolic processes of regulation of the body with signs of overwork in athletes and violations of the mechanisms of regulation of the cardiovascular, central nervous, and endocrine systems with signs of pronounced stress (most often debilitating) (catabolism in muscles and liver; Figure 1).

The second class was characterized by a slight predominance of catabolic and anabolic processes of body regulation with signs of fatigue in athletes. In this group, there was a depletion of neurohumoral mechanisms of regulation and a decrease in the functional reserves of the body by half compared to that during the optimal state of health. During stress testing, a one-and-a-half times decrease in the functional performance of the body was recorded compared to the optimal level of the functional state of the body (catabolism in the muscles and liver; Figure 1).

The third class was characterized by a slight predominance of anabolic over catabolic processes of body regulation, with signs of a satisfactory condition in the examined athletes. This group easily endured intense physical and psychological stress (anabolism in the muscles and liver; Figure 1).

The fourth class was characterized by an absolute predominance of anabolic processes of regulation of the body (anabolism in the muscles and liver; Figure 1). Athletes in this group had a high level of functional reserves, and the mechanisms of metabolic and neuroendocrine interaction of body systems demonstrated optimal regulation.

### 2.3. Analysis of Blood Parameters

Blood sampling from athletes with the highest achievements was carried out strictly on an empty stomach, according to the standard method [16], from 8 to 10 a.m., at the clinical diagnostic laboratory of the State Scientific Center of the Federal Medical and Biological Center named after A.I. A.I. Burnazyan FMBA of Russia.

Vacutainers containing K2EDTA as an anticoagulant were pre-labeled. The resulting biomaterial in a vacuum tube was centrifuged at 3500 rpm, and the supernatant was transferred into pre-labeled polypropylene tubes. Before performing quantitative biochemical analysis, blood plasma samples were frozen at a temperature no higher than −20 °C. Biochemical parameters of blood were studied using the modular platform Cobas 6000 (Roche Diagnostics, Mannheim, Germany). When analyzing peripheral blood, the following indicators were determined: acid phosphatase (U/L), lactate (mmol/L), total protein (g/L), albumin (g/L), creatinine (µmol/L), urea (µmol/L), uric acid (mmol/L), amylase (U/L), triglycerides (mmol/L), total cholesterol (mmol/L), high-density lipoproteins (HDL-mmol/L), total bilirubin (mmol/L), direct bilirubin (mmol/L), alanine aminotransferase (ALAT-Me/L), aspartate aminotransferase (ASAT-Me/L), creatine kinase (Me/L), creatine kinase-MB (Me/L), lactate dehydrogenase (Me/L), gamma-glutamyl transpeptidase (gamma-GT-Me/L), alkaline phosphatase (Me/L), total calcium (mmol/L), phosphorus (mmol/L), magnesium (mmol/L), iron (mmol/L), somatotropic hormone (GH-ng/mL), thyroid stimulating hormone (TSH-Me/l), T4 free (Me/L), prolactin (Me/L), total testosterone (nmol/L), and bone resorption marker (gross-laps -b ng/mL). The results of the laboratory analyses were entered by a senior researcher at the TsSMiR FMBTS im. A. I. Burnazyan into individual cards of athletes passing the IME.

### 2.4. Urine Analysis

Biomaterial sampling was performed in the morning on an empty stomach. Before collecting urine, a thorough examination of the external genital organs was carried out, and an average portion of urine was collected in a sterile container. Pre-labeled polypropylene sample tubes were carefully delivered to the laboratory. Prior to analysis, the biomaterial in these test tubes was quickly frozen at temperatures not higher than −20 °C. Microscopic studies of urine were performed using AXIO microscopes with an Imager A1 video system (Axiostar Plus; Carl Zeiss, Oberkochen, Germany). Physicochemical properties were studied using an Aution Elevan AE-4020 urine analyzer (Arkray Inc., Kyoto, Japan). The results of the laboratory analyses were entered by a senior researcher at the TsSMiR FMBTS im. A. I. Burnazyan into individual cards of athletes passing the IME.

### 2.5. Statistical Analysis

A pairwise comparison of phenotypic classes for each metabolite was performed using Student’s *t*-test with Bonferroni correction. The analysis was performed using [17] URL https://www.R-project.org/ (accessed on 31 August 2022) and the package rstatix [18].

### 2.6. Data Preparation

Before using the data for machine learning models, they were normalized and brought to the same range. The data conversion process consisted of the following steps.

1. All string data, such as “Gender,” “Type of load,” and “Type of sport,” were converted into categorical features.
2. Missing values in the numerical features were replaced by the average of the feature range, and missing values in the categorical features were replaced by the mode of values of this feature.
3. All numerical features were normalized using Z-scaling of the data based on mean and standard deviation (Equation (1)):

(1) $$z=\;\;\frac{x-\mu }{\sigma },$$

where *μ* = mean of the feature, and *σ* = standard deviation

To train and validate the model, the input data were divided into training and validation sets at the following proportions: 60 and 40% for the training and validation set, respectively.

### 2.7. Data Classification Using the Random Forest Algorithm

The total number of decision trees in the model and the maximum depth of trees were set as the regularization parameters. The following metrics were used to evaluate the classification results:

- Overall accuracy (Equation (2)) is defined as the number of correctly predicted items (true positive (TP), true negative (TN)) over total of item to predict (true positive (TP), true negative (TN), false positive (FP) and false negative (FN)).

(2) $$Overall\;accuracy=\frac{\sum_{i=1}^{n}(TP_{i\;}+TN_{i})}{\sum_{i=1}^{n}(TP_{i\;}+TN_{i}+FP_{i}+FN_{i})},\;$$

where *n* is the number of classes (in our case, *n* = 4).

- Average accuracy is defined as the arithmetic mean of all the accuracy scores of different classes (Equation (3)).

(3) $$Average\;accuracy=\frac{Acc_{1}+Acc_{2}+\cdots +Acc_{n}\;}{n},\;$$

where *n* is the number of classes (in our case, *n* = 4) and $Acc_{i\;}\;$ is the accuracy of the $i_{th}$ class, defined as the number of correctly predicted items for each class over the total number of items to predict (Equation (4)).

(4) $$Acc_{i}=\frac{TP_{i}+TN_{i}}{TP_{i}+TN_{i}+FP_{i}+FN_{i}}$$

- Macro-average precision is defined as the arithmetic mean of all the precision scores of different classes (Equation (5)).

(5) $$Macro-average\;precision=\frac{Prec_{1}+Prec_{2}+\cdots +Prec_{n}\;}{n},\;$$

where *n* is the number of classes (in our case, *n* = 4) and $Prec_{i\;}$ is the precision of the $i_{th}$ class, defined as the number of true positives (TP) over the number of true positives plus the number of false positives (FP), as described in Equation (6):

(6) $$Prec_{i}=\frac{TP_{i}}{TP_{i}+FP_{i}}$$

- Macro-average recall is defined as the arithmetic mean of all the recall scores of different classes (Equation (7)).

(7) $$Macro-average\;recall=\frac{Recall_{1}+Recall_{2}+\cdots +Recall_{n}\;}{n},\;$$

where *n* is the number of classes (in our case, *n* = 4) and $Recall_{i\;}$ is the recall of the $i_{th}$ class, defined as the number of true positives (TP) over the number of true positives plus the number of false negatives (FN), as described in Equation (8):

(8) $$Recall_{i}=\frac{TP_{i}}{TP_{i}+FN_{i}}$$

- Micro-average recall is defined as the sum of true positives (TP) for all the classes divided by the actual positives and not the predicted positives (False Negative (FN)), as described in Equation (9):

(9) $$Micro-average\;recall=\frac{TP_{1}+TP_{2}+\cdots +TP_{n}\;}{(TP_{1}+TP_{2}+\cdots +TP_{n})+\left(FN_{1}+FN_{2}+\cdots +FN_{n}\right)},\;$$

where *n* is the number of classes (in our case, *n* = 4).

Microsoft Azure ML Machine Learning Studio was used to implement the random forest model.

The following model regularization parameters were set for training (Table 2).

### 2.8. Classification Using the Multinomial Logistic Regression Algorithm

Multinomial logistic regression is a classification method that generalizes logistic regression to multi-classification problems—that is, problems with more than two possible discrete outcomes. In the multinomial logistic regression model, a binary logistic regression equation was built for each category of the dependent variable. In this case, one of the categories of the dependent variable becomes the reference variable, and all the other categories are compared.

In general, the multinomial logistic regression equation can be written as in Equation (10):

(10) $$p\left(y=c|x;\;\theta \right)=\frac{e^{\theta_{c}^{T}x}}{\sum_{j=1}^{k}e^{\theta_{j}^{T}x}}\;$$

where *x* is the vector of regressors, *y* is the dependent variable taking the values {1, 2,…, *k*}, and *θ* is the regression parameter determined using machine learning methods.

A classification model using multinomial regression was built using tools provided by the MS Azure ML Machine Learning Studio.

In relation to our problem, the regressors *x* represent the normalized features of the original dataset, and the dependent variable *y* takes the value 1…4 *y* = {1, 2, 3, 4}. The optimal regression parameter *θ* was determined using a machine learning studio that uses the gradient descent method to minimize the error function, which can be expressed as (Equation (11)):

(11) $$S\left(\theta \right)=-\frac{1}{m}\sum_{i=1}^{m}\sum_{c=1}^{k}\left[y_{i}=c\right]\;\log p\left(y_{i}=c|x_{i};\theta \right)+\alpha {\left\|\theta \right\|}_{F},\;$$

where *m* is the number of elements in the training set, *k* is the number of classes (in our case, *k* = 4), α is the regularization parameter, and ||*θ*||F is the Frobenius norm of matrix [*θ*1,…, *θ*k].

Blood and urine biochemistry indicators were scored and weighted by the feature importance metric, which was calculated using the following algorithm:

We computed the reference score s of the model (for instance, the accuracy) on the test dataset (D).

For each feature j (column of D):

- For each repetition *k* in 1…*K*:
- Randomly shuffle column of the dataset to generate a corrupted version of the data named ${\tilde{D}}_{k,j}$
- Compute the score $s_{k,j}$ (accuracy) of the model on corrupted data ${\tilde{D}}_{k,j}$
- Compute importance score $i_{j}\;$ for feature $f_{j}$ defined as (Equation (12)):
  (12) $$i_{j}=s-\frac{1}{K}\sum_{k=1}^{K}s_{k,j}$$

Machine learning algorithms were implemented using Microsoft Machine Learning Studio. In order to prevent overfitting, tools for regularization and hyperparameter optimization were used. A package for performing regularization and hyperparameter optimization was provided by the Microsoft Machine Learning Studio, based on the “Cross-validation with a parameter sweep” algorithm. For the decision forest model, the following hyperparameters were regularized:

- Number of decision trees;
- Depth of the tree;
- Number of random splits;
- Number of samples per leaf.

For multinomial regression:

- Optimization tolerance;
- L1 (Lasso) regularization weight;
- L2 (Ridge) regularization weight.

For cross-validation, the dataset was split into 5 consecutive folds.

We have published a project that is available for viewing and studying at https://gallery.azure.ai/Experiment/sports-scoring-DT# (accessed on 31 August 2022).

## 3. Results

### 3.1. Decision Forest and Multinomial Regression Data Classification Models

In total, 3661 healthy and trained athletes were analyzed. The study cohort comprised subjects with the phenotype “catabolism” or “anabolism”, subdivided in four classes (Figure 1) according to the predominant processes occurring in muscle (classes 1 and 2) or in the liver (classes 3 and 4). The properties of classes are described in the subsection “Subjects” (see Section 2).

The random forest algorithm is a universal machine learning algorithm, the essence of which involves using an ensemble of decision trees. A decision tree is a structure in which an inner node represents a feature or attribute, a branch represents a decision rule, and each leaf node represents a result. Using decision rules, each node in the tree separates values such that the classification groups are as different from each other as possible, and the members of each classification group are as similar as possible. A random forest is made up of a large number of individual decision trees that work as an ensemble of methods. Each tree in the random forest returns a class prediction, and the class with the most votes is the forest prediction. 

**Figure 2:**
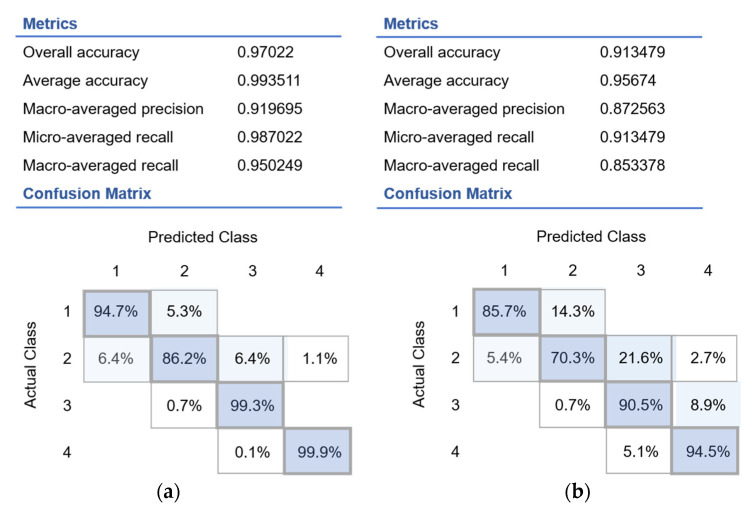
Confusion matrix of decision forest predictive results obtained on the testing dataset (**a**) for the muscle metabolism: Groups 1 and 2 (catabolism), Groups 3 and 4 (anabolism) and (**b**) for the liver metabolism: Groups 1 and 2 (catabolism), Groups 3 and 4 (anabolism). The percentage of recognized files is indicated in the intercept of the different classes, unless the number is in the intercept between different phenotypes.

The proposed classification model showed resistance to noise and achieved high accuracy of up to 0.99, 0.98 recall for muscle metabolism classification, and 0.95 accuracy at 0.85 recall for liver metabolism classification (Figure 2). The model could distinguish between the classes studied. The classification results are presented as a confusion matrix, where each cell is equal to the volume of the *i*-th class to which the properties of the *j*-th class were assigned (Figure 2).

**Figure 3:**
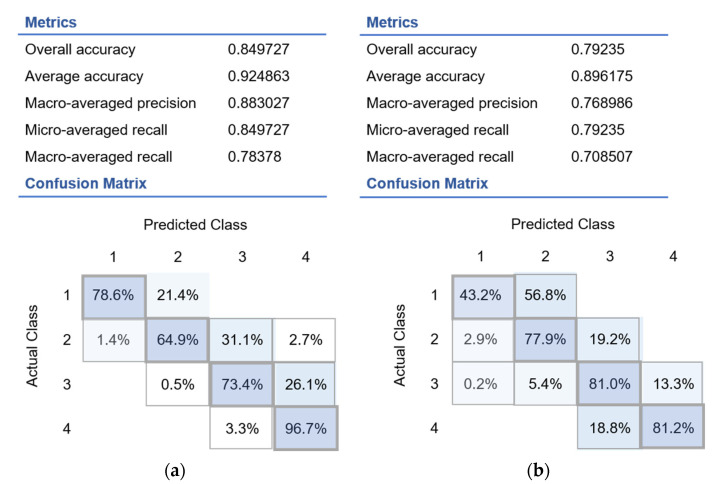
Confusion matrix of multinomial regression predictive results obtained on the testing dataset (**a**) for the muscle metabolism: Groups 1 and 2 (catabolism), Groups 3 and 4 (anabolism) and (**b**) for the liver metabolism: Groups 1 and 2 (catabolism), Groups 3 and 4 (anabolism). The percentage of recognized files is indicated in the intercept of the different classes unless the number is in the intercept between different phenotypes.

Among the data characterizing the health status of 3640 study participants, 60% were enrolled for training, and 40% were used for testing. Over 94% of the data were correctly classified, resulting in a final accuracy of >0.90. For the multinomial regression model, the accuracy, precision and recall parameters were determined for the studied phenotypes, as shown in Figure 3.

Although the multinomial regression model shows acceptable accuracy and response results of up to 0.92 and 0.78, respectively, its performance is noticeably lower than that of the random forest classification model (Figure 3). Therefore, we can conclude that the random forest classification model is more optimal and noise-tolerant for the datasets presented in this study. However, both models show a stable relationship between the parameters of blood biochemistry and the classes that characterize the phenotypes “catabolism” and “anabolism”.

**Figure 4:**
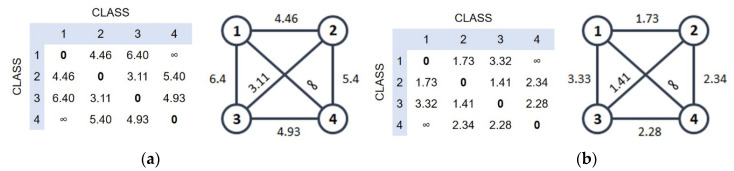
The similarity graph between studied classes constructed using data for decision forest (**a**) and multinomial regression (**b**). Distance between nodes indicates similarity as a measure of the amount of overlap between classes.

We proposed a measure of similarity between the studied classes in a graphical form, in which the nodes indicate the classes, and the edges between the nodes characterize the similarity of the two classes, measured as the Bhattacharya distance (Figure 4).

It is shown that both proposed models can effectively distinguish between the studied classes characterizing the metabolism in muscles and the liver: classes 1 and 2 are characterized as catabolism and classes 3 and 4 are characterized as anabolism (Figure 4). From the results obtained, it should be noted that the classes characterizing the phenotype “anabolism” or “catabolism” are closely related. Thus, the distance between classes 1 and 2 (“catabolism”) for the decision forest and multinomial regression models was only 4.46 and 1.73, respectively. The distance between classes 3 and 4 (“anabolism”) for these models is 4.93 and 2.28, respectively. The most remote according to the classification of class 1, with a predominance of catabolic processes of regulation of the body with signs of overwork of athletes, and class 4, with the absolute predominance of anabolic processes of regulation of the body in athletes, are characterized by the greatest remoteness for both decision forest and multinomial regression models (Figure 4).

### 3.2. Parameters of Blood and Urine Biochemistry as Predictors for the Classification of Phenotypes “Catabolism” and “Anabolism”

The impact of blood and urine biochemistry indicators on the results of the multinomial regression model was accounted for using the permutation feature importance method. Permutation feature importance measures the increase in the prediction error of the model after random shuffling of the feature values, which breaks the relationship between the feature and the true outcome.

**Figure 5:**
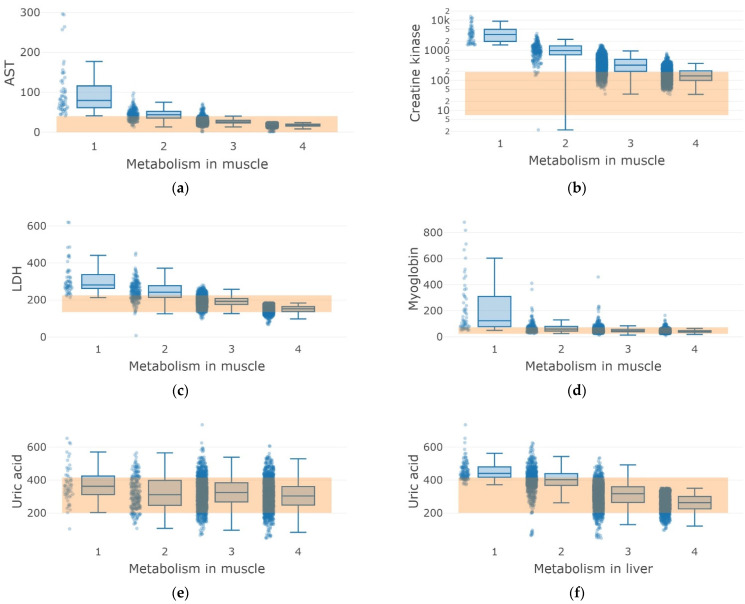

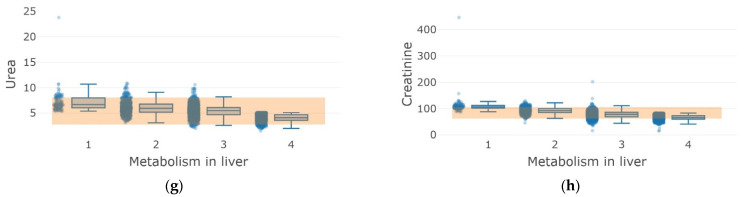
Distribution histograms of urine and blood biochemistry indicators for the studied metabolism in muscles (**a**–**e**) and liver (**f**–**h**) indicating classes (1 and 2 correspond to the “catabolism” phenotype, 3 and 4 correspond to the “anabolism” phenotype), which have the greatest impact on result in multinomial regression model: AST, aspartate aminotransferase (**a**), creatine kinase (**b**), lactate dehydrogenase, LDH (**c**), myoglobin (**d**), uric acid (**e**,**f**), urea (**g**) and creatinine (**h**), The orange background shows the ranges of “reference” values for the presented indicators.

In the segmentation of comparison classes in muscle and liver metabolism (Table 3 and Table 4), it was possible to determine six and three biochemical parameters, respectively, which played a decisive role in classification (Figure 5).

Pairwise comparisons of phenotypic classes for each metabolite were performed using Student’s *t*-test with Bonferroni correction. The phenotypes were significantly different (Supplementary Materials Table S3 and Figure 5). In particular, muscle and liver “catabolism” phenotypes (Classes 1 and 2) were characterized by increased AST (Class 1—99.95 ± 60.35 U/L, grade 2 44, 24 ± 13.22 U/L), CK (Class 1—4092.20 ± 2875.23 U/L), LDG (Class 1—313.21 ± 88.70 U/L) compared with the “anabolism” phenotype: AST (Class 3—27.15 ± 7.23 U/L, Class 4—17.70 ± 3.34 U/L), CK (Class 3—379.55 ± 242.51 U/L, 164.87 ± 92.31 U/L), LDG (Class 3—193.06 ± 27.43 U/L, Class 4—151.41 ± 18.47 U/L) (Figure 5). Myoglobin content increased for a pronounced “catabolism” phenotype (grade 1 222.11 ± 207.26 µg/L) compared with the “anabolism” phenotype and reference values (Figure 5). Acids and creatinine were also elevated amongst athletes with the catabolism phenotype of Class 1 (5.75 ± 1.25 µmol/L for uric acid and 82.38 ± 16.60 µmol/L for creatinine) compared with the reference values and classes of the anabolism phenotype (Class 3—5.32 ± 1.47 µmol/L and Class 4—4.98 ± 1.29 µmol/L for uric acid and Class 3—78.04 ± 18.77 µmol/L for creatinine).

The use of multinomial regression models displayed comparable results in the identification of the most significant indicators for the classification of catabolic and anabolic phenotypes.

## 4. Discussion

The primary reason for the decrease in the physical performance of athletes is the reprogramming of metabolic mechanisms of regulation due to changes in biochemical processes and the work of bioregulation systems of the body, leading to structural disorders. The severity of catabolic or anabolic mechanisms of regulation is determined by the intensity and duration of physical activity. When the function of the adrenal glands is inhibited, the concentration of the products of catabolism of protein and amino acid compounds (hepatic metabolism) increases in the blood, whereas the content of metabolites of lipid peroxidation and nucleic acids increases in the muscle tissue, with a pronounced decrease in the activity of respiratory enzymes, which is extremely important in sports medicine. Increased catabolism in the body of athletes contributes to the development of detoxification system blockade and changes in blood plasma, confirming the violation of metabolic processes in the muscle tissues and internal organs.

Predicting the mechanisms of the predominance of anabolic or catabolic processes in the athlete’s body, both at the current time and its change in the dynamics of a four-year macrocycle, is a paramount task in sports medicine and planning the recovery phase of an athlete. In this study, clinical data consist of a large set of heterogeneous features. Such an enormous number of biochemical indicators, most of which are beyond the acceptable ranges, complicates decision-making by physicians. It is compulsory to establish the most significant contributing indicators that display successful post-competitive recovery and performance.

The proposed approach involving classification algorithms is completely impartial and capable of enabling the accurate monitoring of catabolism and anabolism to maintain the effective management of the training activities of athletes. The most significant biochemical indicators can be gleaned through the relatively short calculating time with utmost accuracy. Among the machine learning algorithms suitable for multiclass classification problems, the decision forest algorithm is one of the most popular tools. This algorithm provides the ability to process binary, categorical and numerical features. In this case, a simple preliminary preparation of the input data is sufficient, with no scaling or transformation. Calculations can be parallelized into several processes, which significantly reduces the calculation time. The decision forest method is suitable for multidimensional data because it operates with subsets. The method is robust to data outliers and indifferent to non-linear behavior of features due to the balancing of errors in imbalanced class sets, which allows us to reduce the overall error rate. Eventually, each decision tree has high variance at a low bias. Averaging all the trees in the random forest averages the variance, resulting in a model with low bias and moderate variance.

According to the model indicators (presented in Section 3), the decision forest algorithm showed the best results. However, the multinomial regression algorithm can also reveal the significant features that contribute to the successful recovery of elite athletes. The predominance of anabolic processes amongst examined individuals indicates an excellent functional state and good adaptive reserves of the body, sufficient to overcome intense and prolonged physical exertion. In connection with these aforementioned factors, this study, dedicated to the search for new informative predictors that characterize athletes’ homeostasis, is still relevant at present. To identify the most significant contributing predictors and to evaluate the prevalence of anabolic or catabolic processes, dozens of biochemical indicators (see Section 2.3 and Section 2.4; and Supplementary Materials Table S1) were collected and subsequently included in the model.

The phenotype “catabolism” for muscle and liver metabolism (classes 1 and 2) was characterized by the increase in aspartate aminotransferase (AST), creatine kinase, lactate dehydrogenase (LDH), and myoglobin compared to the phenotype “anabolism” (Figure 5). The reference ranges of blood and urine biochemistry for all classes correspond to the observations regarding study participants’ uric acid, urea, and creatinine. Class 1 was characterized by the absolute predominance of catabolic processes with signs of overwork of athletes, such as violations of the mechanisms of regulation of the cardiovascular, central nervous, and endocrine systems, with remarkably explicit stress (most often debilitating). Meanwhile, Class 2 was undoubtedly inclined toward catabolic processes with signs of fatigue in athletes, the depletion of neurohumoral regulatory mechanisms, and a two-fold decrease in recovery compared with the optimal performance.

A significant increase in the levels of the liver function indicators (AST and ALT), LDH, creatine kinase, and myoglobin in the blood has been shown in athletes under fatigue conditions seven days after intensive exercise (*p* < 0.01) [19,20,21,22]. Indeed, transaminitis in athletes is often mediated by damaged muscle tissue, as demonstrated in this study. The level of liver function indicators can be increased by an order of magnitude compared to the reference values [23,24]. Refusal of intensive training restores the liver function indicators to normal level, in favor of damage to muscle tissue in athletes, a significant increase in correlation with creatine kinase levels for the anabolic phenotype (classes 1 and 2, Figure 5b). However, liver injury factors cannot be completely ignored, because the level of alkaline phosphatase is elevated by 17% (Supplementary materials Table S1).

Almost 70% of the examined athletes participated in minor or moderate exercise, regardless of their phenotype (anabolism or catabolism), whereas the other 30% retained a high intensity of physical activity. We assumed that anabolism under the conditions of moderate physical activity reflects the delayed normalization of indicators after an intense period of exercise, which exceeds 11 days under conditions of overwork [25].

The pronounced catabolism phenotype stands is characterized by meaningfully elevated myoglobin, which is linked to myalgia and extremely negative rhabdomyolysis, closely related to the intensity and duration of physical activity. This condition is characterized by the destruction of the integrity of skeletal muscles after physical activity [26,27]. Furthermore, we determined the increased levels of acids and creatinine in the catabolism phenotype athletes, which is generally acceptable after exercise [28]. We assume that this effect is caused by the offset mechanisms after exercise-mediated energy depletion, when a decrease in the ATP/ADP ratio triggers boosted purine synthesis and the degradation and elimination of adenine nucleotides [28].

## 5. Conclusions

Monitoring blood biomarkers in athletes makes it possible to neutralize negative effects in the post-competitive period by adjusting diet, training load, and recovery strategy. In this regard, population-based studies of athletes are important to assess the effectiveness of the recovery strategy in accordance with clinical reference values and between the phenotypes “anabolism” (recovery state of the body) and “catabolism” (stressed state and overwork as a result of physical exertion).

We introduced the decision forest and multinomial regression models to locate a pattern of the most significant indicators of blood and urine biochemistry affecting the performance of recovery processes in the post-competitive period in athletes.

This study demonstrates that laboratory test values are outside the generally accepted reference limits because they are calculated for a sedentary population and reflect adaptations to regular and prolonged physical activity. We also demonstrated that muscle-related metabolites (AST, CK, LDH, and ALT levels, and indicators of the ornithine cycle, such as creatinine, urea acid, and urea) are significant indicators for the classification of the “catabolism” and “anabolism” phenotypes. The present model for the stratification of prevalence between catabolism and anabolism needs further adjustment. Although the proposed model efficiently discriminates a huge number of biochemical indicators and is capable of establishing the most significant contributing factors, some factors, such as gender, demonstrate less significant input to distinguish these phenotypes. This may limit and affect further consideration of the final decision in adjusting the post-competitive recovery to achieve efficient athlete performance.

## Figures and Tables

**Table 1:** Anthropometric characteristics.

Kind of Sport	Number of Athletes	Gender Ration, % (M/F)	Age (Mean), Years Old	SD	Phenotype “Anabolism”		Phenotype “Catabolism”	
					Group 1 Muscle (Classes 1 and 2)	Group 2 Liver (Classes 3 and 4)	Group 3 Muscle (Classes 1 and 2)	Group 4 Liver (Classes 3 and 4)
endurance	823	59/41	21, 35	6, 73	763	663	60	160
psycho-emotional	137	57/43	23, 61	10, 99	136	102	1	35
strength endurance	479	63/37	20, 21	5, 55	431	356	48	123
speed endurance	225	60/40	21, 22	5, 82	209	162	16	63
speed-power	479	60/40	21, 14	6, 60	442	327	37	152
difficult coordination	165	45/55	19, 04	4, 86	138	142	27	23
technical	1353	56/44	21, 35	7, 08	1261	1059	92	294
Total	3661	58/42	21, 15	6, 83	3380	2811	281	850

Data on anthropometric characteristics, biochemical parameters of blood and urine are presented in the Supplementary Materials Table S1.

**Table 2:** Regularization parameters of the random forest model.

Number of Decision Trees	Maximum Depth of the Tree	Number of Random Splits	Minimum Number of Samples per Leaf
64	128	128	128

**Table 3:** Features with the highest impact in the random forest model (importance score > 0.1).

Metabolism in Muscles		Metabolism in the Liver	
Feature	Score	Feature	Score
Creatine phosphokinase (CFK)	0.95	Creatinine	0.35
Lactate dehydrogenase (LDH)	0.74	Urea acid	0.32
Aspartate aminotransferase (AST)	0.63	Urea	0.22

Complete data on the weight values of blood and urine biochemistry parameters are presented in Supplementary Materials, Table S2.

**Table 4:** Features with the highest impact in the multinomial regression model (importance score > 0.1).

Metabolism in Muscles		Metabolism in the Liver	
Feature	Score	Feature	Score
Aspartate aminotransferase (AST)	0.57	Uric acid	0.46
Creatine kinase (CK)	0.50	Urea	0.32
Lactate dehydrogenase (LDH)	0.43	Creatinine	0.19
Alanine aminotransferase (ALT)	0.29	Sex (F)	0.16
Myoglobin	0.29		
Uric acid	0.14		

Complete data on the weight values of blood and urine biochemistry parameters are presented in Supplementary Materials, Table S2.

## Data Availability

Not applicable.

## Supplementary Materials

The following supporting information can be downloaded at: https://www.mdpi.com/article/10.3390/sports10100160/s1, Table S1: Data on anthropometric characteristics, biochemical parameters of blood and urine; Table S2: Data on the weight values of blood and urine biochemistry parameters; Table S3: Pairwise comparisons of phenotypic classes for each metabolite.
